# Quality of low‐carbohydrate diets among Australian post‐partum women: Cross‐sectional analysis of a national population‐based cohort study

**DOI:** 10.1111/mcn.13502

**Published:** 2023-03-20

**Authors:** Sophie Lewandowski, Elizabeth Neale, Ellie D'Arcy, Allison M. Hodge, Danielle A. J. M. Schoenaker

**Affiliations:** ^1^ School of Medical, Indigenous and Health Sciences University of Wollongong Wollongong New South Wales Australia; ^2^ Illawarra Health and Medical Research Institute Wollongong New South Wales Australia; ^3^ Integrated Care Western New South Wales Local Health District New South Wales Dubbo Australia; ^4^ Cancer Epidemiology Division Cancer Council Victoria Melbourne Victoria Australia; ^5^ Melbourne School of Population and Global Health University of Melbourne Parkville Victoria Australia; ^6^ School of Primary Care, Population Sciences and Medical Education University of Southampton Southampton UK; ^7^ NIHR Southampton Biomedical Research Centre University of Southampton and University Hospital Southampton NHS Foundation Trust Southampton UK

**Keywords:** carbohydrate quality, gestational diabetes, low‐carbohydrate diet, post‐partum, weight loss

## Abstract

Low‐carbohydrate diets (LCDs) are popular among people attempting weight loss and recommended for pregnant women with gestational diabetes (GDM), but they may increase health risks if nutritionally inadequate. We aimed to describe the dietary intake of post‐partum women according to their relative carbohydrate intake, overall, and among women attempting weight loss or diagnosed with GDM in their recent pregnancy. This cross‐sectional population‐based cohort study included 2093 post‐partum women aged 25–36 years who participated in the Australian Longitudinal Study on Women's Health. Dietary intake was assessed using a validated food frequency questionnaire. Relative carbohydrate intake was determined using a previously developed LCD score. Data were weighted to account for oversampling of women from rural/remote areas. More than half of women (*n*[weighted] = 1362, 66.3%) were trying to lose weight, and 4.6% (*n*[weighted]=88) had GDM in their recent pregnancy. Women with the lowest relative carbohydrate intake (LCD score quartile 4) consumed 36.8% of total energy intake from carbohydrates, and had a lower intake of refined grains, whole grains, fruit and fruit juice, and a higher intake of red and processed meat, compared with women with the highest relative carbohydrate intake (quartile 1). Different food groups, both healthy and unhealthy, were restricted depending on whether women were attempting weight loss and had recent GDM. These findings may reflect a lack of knowledge among post‐partum women on carbohydrates and dietary guidelines. Health professionals may have an important role in providing advice and support for post‐partum women who wish to restrict their carbohydrate intake, to ensure optimal diet quality.

## Key messages

- Low‐carbohydrate diets have become increasingly popular, but they may increase health risks if nutritionally inadequate.
- In a national population‐based study of Australian post‐partum women, relatively low carbohydrate intake was associated with healthy and unhealthy food choices, including consumption of less refined grains and fruit juice, but also less whole grains and fruit and more red meat and processed meat.
- Given the critical role of carbohydrate quantity and quality in disease prevention, health professionals should inform and support women who wish to restrict their carbohydrate intake to consume a balanced diet in line with dietary guidelines.

## 1 INTRODUCTION

Carbohydrate restriction has received much interest and become increasingly popular in the past decade (International Food Information Council, 2018, 2019; Malik & Hu, 2007). While there is no agreed definition of a low‐carbohydrate diet (LCD), dietary guidelines typically recommend consuming 45%–65% of total energy from carbohydrates (National Health and Medical Research Council, 2013; US Department of Agriculture, 2020). A diet that includes a lower proportion of energy from carbohydrates (<45%) can be considered an LCD or reduced‐carbohydrate diet (Hu et al., 2012; Oh et al., 2022). In the short term, LCDs may lead to greater weight loss compared with low‐kilojoule and balanced‐carbohydrate diets, and improve glucose control in adults with obesity and diabetes (Churuangsuk et al., 2018; van Zuuren et al., 2018). However, findings from intervention studies with longer‐term follow‐up (1–2 years or more after the intervention) show minor differences in weight loss and no differences in other cardiometabolic risk markers (Avenell et al., 2004; Hu et al., 2012; Naude et al., 2022). Despite the lack of scientific evidence on their sustained health benefits, LCDs are popular among the public with up to 20% of people in high‐income countries reporting they have tried or followed an LCD (International Food Information Council, 2019), potentially fuelled by media stories and social norms (Churuangsuk et al., 2020; Hawkins et al., 2020).

Specific population groups may be more likely to consume an LCD, including post‐partum women. Post‐partum women are advised to consume a diet in line with national dietary guidelines for women of reproductive age, with higher intakes of vegetables and whole grains recommended for women who are breastfeeding (National Health and Medical Research Council, 2013; US Department of Agriculture, 2020). Optimal diet quality is important for post‐partum women to enhance recovery from pregnancy and childbirth, support nutrition requirements for breastfeeding and prevent adverse health conditions such as obesity and noncommunicable disease (National Health and Medical Research Council, 2013; US Department of Agriculture, 2020). Compared with the general population, post‐partum women may be more likely to restrict their carbohydrate intake for weight loss reasons including reducing post‐partum weight retention (PPWR) and maintaining or achieving a healthy weight in preparation for a subsequent pregnancy, despite a lack of evidence and recommendations on the effectiveness of an LCD for post‐partum weight loss (Chen et al., 2021; Schoenaker et al., 2020). Additionally, limiting carbohydrate intake is often recommended during pregnancy for women who are diagnosed with gestational diabetes mellitus (GDM) to maintain optimal glucose control (Kapur et al., 2020). After pregnancy, women with recent GDM are advised to eat healthily in line with general dietary guidelines to reduce their risk of type 2 diabetes (D'Arcy et al., 2020); however, limited evidence suggests these women may continue to restrict their carbohydrate intake after pregnancy (Tang et al., 2021). Restricting carbohydrates below the recommended intake may compromise the recovery and future health of post‐partum women.

Diets that are relatively low in carbohydrates are higher in fat and protein, and controversy remains as to whether consumption of LCDs, in particular with high consumption of animal fat and protein, is related to potentially adverse cardiovascular and metabolic health effects (Bao et al., 2016; Chen et al., 2021; Ludwig et al., 2018; Rayner et al., 2020). LCDs may also be nutritionally inadequate if they eliminate core food groups such as fruit and grains. The quality of carbohydrates and types of foods consumed as part of an overall diet, in addition to the quantity of carbohydrates, are critical in influencing health and disease risks (Ludwig et al., 2018).

We are not aware of previous population‐based studies that have looked at the dietary intake of post‐partum women who have a relatively low carbohydrate intake, in particular post‐partum women who are trying to lose weight or who were diagnosed with GDM in their recent pregnancy. Given the importance of optimal diet quality for post‐partum women, these insights could inform public health messages and advice on consuming a balanced diet.

The aims of this study were therefore to use data from a national population‐based cohort study to describe the dietary intake of post‐partum women according to their relative carbohydrate intake, including among subgroups of women attempting weight loss and women diagnosed with GDM in their recent pregnancy.

## 2 METHODS

### 2.1 Study population

The Australian Longitudinal Study on Women's Health (ALSWH) is an ongoing longitudinal population‐based study designed to provide an evidence base for the development and evaluation of policies and practice guidelines that affect women. The study was initiated in 1996 and 14,247 women were recruited into the 1973–1978 cohort at age 18–23 years. Participants were sampled at random from the national Medicare health insurance database, which includes all Australian citizens and permanent residents. Women living in rural and remote areas were intentionally oversampled. At baseline, the study sample was broadly representative of the general population of women of the same age based on census data on socio‐demographic characteristics. The study includes more women in married or de facto relationships compared with the general population (20.3% vs. 11.4%), and women in the workforce are slightly underrepresented (60.6% vs. 73.7%) (Brown et al., 1998). Nonresponse over time has been inevitable, especially due to not being able to contact women for follow‐up surveys; however, retention since baseline has had minimal impact on representativeness (Powers & Loxton, 2010). Further details on recruitment, retention and survey methods have been published previously (Brown et al., 1998; Dobson et al., 2015; Lee et al., 2005) and can be found online at http://www.alswh.org.au/.

After the first survey in 1996, women completed surveys every 3–4 years. For the current study, data were taken from the 2003 questionnaire (Survey 3, age 25–30 years) or the 2009 questionnaire (Survey 5, age 31–36 years) when validated dietary intake data were collected. Of the 10,396 women who completed Survey 3 and/or Survey 5, women who reported a live birth in the previous 12 months and were not currently pregnant were included in this study (*N* = 2093). Survey 3 data were included (*n* = 944), or Survey 5 data if women were not already included based on Survey 3 data (*n* = 1149).

### 2.2 Dietary assessment

Dietary intake was assessed using the Dietary Questionnaire for Epidemiological Studies (DQES) version 2, a food frequency questionnaire (FFQ) developed by the Cancer Council Victoria for Australian adults (Ireland et al., 1994). This FFQ has been validated against 7‐day food records, showing energy‐adjusted Pearson correlation coefficients of 0.70 for carbohydrates, 0.68 for fats and 0.32 for proteins (Hodge et al., 2000). Using a 10‐point scale ranging from ‘never’ to ‘3 or more times per day’, participants were asked to report their usual frequency of food and beverage intake over the last 12 months including individual food items from core food groups. Portion size data were collected based on photos. Nutrient intakes were calculated using the 1995 National Reference Food Composition Database of Australian foods (NUTTAB95) (Lewis et al., 1995). The glycaemic index (GI) of individual food items was calculated using the 2002 International table of GI and glycaemic load (GL) values (Hodge et al., 2004). The GL was calculated by multiplying the GI with carbohydrate intake (in g) from each food item and summing across items. The average GI was calculated by dividing the GL by the total carbohydrate intake (in g) and did not include alcohol consumption (Hodge et al., 2004). Consumption of individual foods was converted from g/day to serves/day in line with the 2013 Australian Dietary Guidelines (National Health and Medical Research Council, 2013), and foods were grouped to report intake of food groups such as whole grains, refined grains, dairy, red meat, processed meat and discretionary foods (Supporting Information: Table 1).

### 2.3 LCD score

A previously developed and commonly utilised LCD score (Bao et al., 2016; Halton et al., 2008; Looman et al., 2018) was used to determine the carbohydrate content of an individual's diet relative to their protein and fat intake. Participant's proportion of total energy intake from carbohydrates, fats and proteins was split into 11 quantiles from lowest to highest intake and given a score ranging from 10 to 0 for carbohydrates and 0 to 10 for fats and proteins. These components were summed to give an overall LCD score ranging from 0 to 30, with a higher LCD score representing a relatively lower carbohydrate and higher fat and protein intake. LCD scores were analysed as quartiles, with quartile 1 representing a diet with the least carbohydrate restriction (i.e., highest carbohydrate content), and quartile 4 representing a diet with the most carbohydrate restriction (i.e., lowest carbohydrate content).

### 2.4 Assessment of attempted weight loss

Self‐reported data on whether women were on a diet to lose weight within the previous 12 months were collected at Surveys 3 and 5. The question included in Survey 3 was ‘How often have you gone on a diet (that is, limiting how much you ate) in order to lose weight during the last year?’, with response options ranging from ‘Never’ to ‘I am always trying to lose weight’. In Survey 5, the question was ‘Have you used any of these methods to lose weight or to control your weight or shape in the last twelve months?’, with options including different weight loss strategies such as ‘Commercial weight loss programs’ and ‘Low glycaemic index diet’. For analysis, the data were categorised as yes or no to reflect whether weight loss had been attempted during the previous 12 months or not.

### 2.5 Assessment of GDM

From Survey 4 onwards, women were asked to report pregnancy outcomes and dates of birth for all their live‐born children (including children born before Survey 4). These data were used to determine the timing and outcome of pregnancies in the year before baseline. For each pregnancy resulting in a live birth, women were asked ‘Were you diagnosed with or treated for gestational diabetes?’. The 1998 ADIPS criteria for GDM diagnosis were used at the time of the study (Hoffman et al., 1998). A reliability study among a subgroup of women from New South Wales, Australia (*n* = 1914) demonstrated a high agreement of 91% between self‐reported GDM diagnosis in the study and administrative data records (Gresham et al., 2015).

### 2.6 Assessment of participant's characteristics

Self‐reported data were collected on age, country of birth, area of residence (remoteness), highest qualification completed, ability to manage on available household intake, smoking status, alcohol consumption, number of children, time since childbirth (based on the date of survey completion and date of most recent live birth), breastfeeding status and self‐reported doctor‐diagnosed diabetes. Body mass index (BMI) was calculated based on self‐reported height and weight (kg/m^2^) and categorised as normal weight (BMI < 25.0 kg/m^2^), overweight (BMI = 25.0–29.9 kg/m^2^) and obesity (BMI ≥ 30.0 kg/m^2^). Only 2% of women reported a BMI < 18.5 kg/m^2^ and were therefore included in the normal weight group. Physical activity scores were derived from validated questions on frequency and duration of walking (for recreation or transport) and reported information on moderate‐ and vigorous‐intensity physical activity in the last week. The level of physical activity was categorised as sedentary/low (<600 metabolic equivalents of task [MET]‐min/week) or moderate/high (≥600 MET‐min/week) (Brown et al., 2008).

### 2.7 Statistical analysis

Data were weighted to account for area of residence. All statistical analyses were conducted using Stata version 17.0 using weighted data (Stata commands svyset and svy). Participant's characteristics and dietary intake were compared across quartiles of LCD scores, and between women who were and were not attempting weight loss, and did or did not have GDM in their recent pregnancy. The number of participants and percentages were presented for categorical variables, and the mean with standard deviation was presented for normally distributed continuous variables or median with interquartile range for nonnormally distributed continuous variables. To estimate statistical differences across categories of LCD quartiles, attempting weight loss and GDM, (multinomial) logistic regression was used for categorical variables (e.g., participant characteristics) and linear regression was used for continuous variables (e.g., dietary intake). Log‐transformed dietary intake variables were used in linear regression for variables that were non‐normally distributed.

### 2.8 Ethical statement

This study was conducted according to the guidelines laid down in the Declaration of Helsinki, and the ALSWH has ongoing ethical approval from the Human Research Ethics Committees of the Universities of Newcastle and Queensland (approval numbers H‐076‐0795 and 2004000224, respectively). All participants provided written consent to join the study and have been free to withdraw or suspend their participation at any time with no need to provide a reason.

## 3 RESULTS

### 3.1 Study population

Post‐partum women in this study (*N*[weighted] = 2059) gave birth on average 6 months before survey completion (SD = 3.4) and had a mean age of 31 years (SD = 3.1). About one‐third (36.1%) had their first live birth in the past 12 months, 4.6% were diagnosed with GDM during their recent pregnancy and 53.2% were currently breastfeeding. Nearly half of the women (45.5%) had overweight or obesity, and 66.3% reported they were trying to lose weight (61.3%, 73.1% and 81.6% of women with normal weight, overweight and obesity, respectively).

Compared with women with a diet relatively high in carbohydrates (LCD score quartile 1), women with low relative carbohydrate intake (LCD score quartile 4) were more likely to be trying to lose weight (72.2% vs. 64.1%), have obesity (22.0% vs. 12.7%), type 1 or 2 diabetes (3.5% vs. 1.4%) and GDM in the most recent pregnancy (6.3% vs. 3.3%), and less likely to be currently breastfeeding (42.9% vs. 56.3%) (Table 1).

**Table 1:** Characteristics of post‐partum women according to quartiles of low ‐carbohydrate‐diet score, *N*[weighted] = 2059^a^.

Characteristics	Low carbohydrate‐diet score				*p* Value^b^
	Quartile 1 (least carbohydrate restriction), *N*[weighted] = 657 (31.9%)	Quartile 2, *N*[weighted] = 484 (23.5%)	Quartile 3, *N*[weighted] = 450 (21.9%)	Quartile 4 (most carbohydrate restriction), *N*[weighted] = 468 (22.7%)	
Age (years), mean (SD)	30.9 (3.1)	31.3 (3.2)	31.5 (3.1)	31.5 (3.2)	0.01
Country of birth, *n* (%)					0.23
Australia	596 (91.2)	456 (94.7)	418 (93.8)	435 (94.1)	
Overseas	57 (8.8)	25.7 (5.3)	27.4 (6.2)	27.3 (5.9)	
Area of residence, *n* (%)					0.15
Urban	404 (61.6)	289 (59.7)	263 (58.4)	250 (53.4)	
Rural or remote	253 (38.4)	195 (40.3)	187 (41.6)	218 (46.6)	
Highest qualification completed, *n* (%)					0.13
No formal or (high) school certificate	128 (19.7)	101 (21.4)	98 (22.1)	120 (26.1)	
Trade/diploma	164 (25.2)	117 (25.0)	114 (25.7)	123 (27.0)	
(Higher) university degree	359 (55.1)	252 (53.6)	231 (52.2)	214 (46.9)	
Ability to manage on income, *n* (%)					0.05
Impossible/difficult all of the time	71 (10.8)	50 (10.4)	54 (12.2)	68 (14.6)	
Difficult sometimes/not too bad	479 (73.0)	352 (73.0)	318 (71.5)	340 (73.0)	
It is easy	107 (16.2)	80 (16.6)	72.6 (16.3)	57.8 (12.4)	
Smoking status, *n* (%)					0.10
Never smoker	418 (63.8)	284 (58.7)	278 (62.0)	257 (55.0)	
Ex‐smoker	173 (26.4)	153 (31.6)	119 (26.6)	145 (30.8)	
Current smoker	64 (9.8)	47 (9.7)	51 (11.4)	66 (14.2)	
Physical activity, *n* (%)					0.59
Low/sedentary (<600 MET‐min/week)	398 (61.8)	299 (62.4)	277 (62.9)	271 (59.7)	
Moderate/high (≥600 MET‐min/week)	246 (38.2)	180 (37.6)	163 (37.1)	183 (40.3)	
Alcohol consumption, *n* (%)					0.06
Nondrinker	106 (16.3)	65 (13.5)	47 (10.5)	58 (12.3)	
Drinker	545 (83.7)	416 (86.5)	402 (89.5)	410 (87.7)	
Body mass index (kg/m^2^), mean (SD)	24.9 (4.9)	25.1 (4.5)	25.5 (5.4)	26.3 (5.5)	0.13
Body mass index classification, *n* (%)					0.04
Normal weight (<25 kg/m^2^)	358 (57.9)	265 (58.3)	248 (58.0)	207 (47.0)	
Overweight (25–<30 kg/m^2^)	182 (29.4)	118 (26.0)	107 (25.0)	137 (31.0)	
Obesity (≥30 kg/m^2^)	78 (12.7)	72 (15.7)	72 (17.0)	97 (22.0)	
Attempting to lose weight, *n* (%)	421 (64.1)	298 (61.7)	305 (68.1)	338 (72.2)	0.03
Number of children, *n* (%)					0.03
One child	222 (33.8)	191 (39.4)	171 (38.0)	180 (38.3)	
Two or more children	435 (66.2)	293 (60.6)	279 (62.0)	289 (61.7)	
Time since birth most recent child					0.01
Up to 6 months	289 (44.0)	234 (48.4)	228 (50.6)	263 (56.2)	
6–12 months	368 (56.0)	250 (51.6)	222 (49.4)	205 (43.8)	
GDM in most recent pregnancy, *n* (%)	20.4 (3.3)	28 (6.0)	14 (3.3)	27 (6.3)	0.04
Breastfeeding status (currently breastfeeding), *n* (%)	358 (56.3)	267 (56.4)	240 (55.6)	190 (42.9)	0.02
Type 1 or 2 diabetes, *n* (%)	9 (1.4)	10 (2.1)	7 (1.6)	16 (3.5)	0.004

Abbreviations: GDM, gestational diabetes; MET, total metabolic equivalent; SD, standard deviation.

^a^ Number of participants differs due to missing data (from *N*[weighted] = 2059 for age and low carbohydrate score to *N*[weighted] = 1911 for GDM in the most recent pregnancy).

^b^ *p* Value from linear regression or (multinomial) logistic regression.

### 3.2 Dietary intake according to relative carbohydrate intake

A diet relatively low in carbohydrates was characterised by a substantially lower proportion of energy from carbohydrates (49% vs. 37% for LCD score quartile 1 vs. quartile 4) and a higher proportion of energy from total fat (33% vs. 42%), while the proportion of energy from protein differed to a lesser extent (18% vs. 22%) (Table 2). A diet relatively low in carbohydrates was further characterised by lower GL and lower daily intake of whole grains (−0.6 serves), refined grains (−0.4 serves), fruit (−0.6 serves), fruit juice (−0.5 serves) and dietary fibre (−3.1 g/MJ) and a higher daily intake of total energy (+505 kJ), saturated fat (+3.8 energy %), red meat (+0.8 serves) and processed meat (+0.3 serves), when comparing LCDs score quartile 1 with quartile 4 (Table 2).

**Table 2:** Dietary intake of post‐partum women according to quartiles of low carbohydrate‐diet score, *N*[weighted] = 2059.

Dietary intake^a^	Low carbohydrate‐diet score				*p* Value^b^
	Quartile 1 (least carbohydrate restriction), *N*[weighted] = 657 (31.9%)	Quartile 2, *N*[weighted] = 484 (23.5%)	Quartile 3, *N*[weighted] = 450 (21.9%)	Quartile 4 (most carbohydrate restriction), *N*[weighted] = 468 (22.7%)	
Low carbohydrate‐diet score, mean (SD)	6.7 (2.6)	13.1 (1.4)	17.9 (1.4)	24.4 (2.7)	<0.0001
Carbohydrate‐related nutrients and foods					
Carbohydrate (energy %), mean (SD)	48.9 (3.4)	44.0 (1.2)	41.2 (0.9)	36.8 (3.1)	<0.0001
Protein (energy %), mean (SD)	18.1 (2.4)	19.4 (2.6)	19.9 (2.7)	21.9 (2.9)	0.0002
Total fat (energy %), mean (SD)	33.4 (4.3)	36.9 (3.4)	39.2 (3.1)	41.7 (2.8)	0.0002
Fibre (g/MJ), mean (SD)	22.5 (8.2)	21.7 (8.2)	21.1 (7.4)	19.4 (7.3)	0.01
Glycaemic index, mean (SD)	52.7 (3.8)	52.5 (3.8)	52.0 (3.9)	51.3 (4.1)	0.004
Glycaemic load, mean (SD)	116.5 (41.2)	109.1 (38.1)	104.6 (36.0)	91.4 (38.9)	0.01
Whole grains (serves), median (IQR)	2.4 (1.3–3.6)	2.3 (1.1–3.4)	2.3 (1.3–3.4)	1.8 (0.8–2.8)	0.01
Refined grains (serves), median (IQR)	1.8 (1.0–2.8)	1.6 (0.9–2.8)	1.5 (1.0–2.6)	1.4 (0.8–2.4)	0.04
Fruit juice (serves), median (IQR)	0.7 (0.1–1.4)	0.4 (0.1–1.2)	0.3 (0.1–0.9)	0.2 (0.1–0.8)	0.03
Fruit (serves), median (IQR)	1.6 (0.8–2.2)	1.0 (0.7–1.8)	1.0 (0.6–1.8)	1.0 (0.6–1.6)	0.03
Vegetables (serves), mean (SD)	2.1 (1.0)	2.2 (1.0)	2.1 (1.0)	2.2 (1.0)	0.09
Discretionary foods (serves), median (IQR)	2.8 (1.7–4.3)	2.9 (1.9–4.3)	3.1 (2.0–4.6)	2.8 (1.9–4.0)	0.05
Other nutrients and foods					
Total energy intake (kJ), mean (SD)	7554 (2530)	7860 (2604)	8041 (2632)	8059 (3244)	0.17
Saturated fat (energy %), mean (SD)	14.1 (2.8)	15.7 (2.7)	16.8 (2.7)	17.9 (2.5)	0.0002
Monounsaturated fat (energy %), mean (SD)	0.3 (0.1)	0.3 (0.1)	0.4 (0.1)	0.4 (0.1)	0.0001
Polyunsaturated fat (energy %), mean (SD)	4.8 (1.6)	5.1 (1.7)	5.3 (1.6)	5.4 (1.7)	0.01
Dairy (serves), mean (SD)	1.9 (0.8)	2.0 (0.8)	1.9 (0.8)	1.9 (0.9)	0.39
Fish (serves), median (IQR)	0.1 (0.1–0.2)	0.1 (0.1–0.2)	0.1 (0.1–0.2)	0.1 (0.1–0.2)	0.003
Red meat (serves), median (IQR)	0.6 (0.3–1.0)	1.0 (0.6–1.4)	1.1 (0.8–1.6)	1.4 (1.0–2.1)	0.01
Processed meat (serves), median (IQR)	0.2 (0.1–0.4)	0.3 (0.2–0.5)	0.4 (0.2–0.6)	0.5 (0.3–0.7)	0.003

Abbreviations: IQR, interquartile range; SD, standard deviation.

^a^ Food group serving sizes are as per the 2013 Australian Dietary Guidelines (see Supporting Information: Table 1).

^b^ *p* Values from linear regression.

#### 3.2.1 Women attempting weight loss

Distributions of dietary intake across quartiles of LCD scores were generally similar for women who were and were not attempting weight loss, except for refined grains and fruit (Table 3). Women attempting to lose weight who restricted their carbohydrate intake the most compared with the least had a substantially lower intake of refined grains (−0.5 serves) and fruit (−0.7 serves), while intake of these foods did not differ according to LCD score among women not attempting weight loss.

**Table 3:** Dietary intake of post‐partum women who are and are not attempting weight loss according to low carbohydrate‐diet score, *N*[weighted] = 2056.

Dietary intake^a^	Women attempting weight loss, *N*[weighted] = 1362			Women not attempting weight loss, *N*[weighted] = 694		
	Quartile 1 (least carbohydrate restriction), *N*[weighted] = 421 (30.9%)	Quartile 4 (most carbohydrate restriction), *N*[weighted] = 338 (24.8%)	*p* Value^b^	Quartile 1 (least carbohydrate restriction), *N*[weighted] = 236 (34.0%)	Quartile 4 (most carbohydrate restriction), *N*[weighted] = 130 (18.8%)	*p* Value^b^
Low carbohydrate‐diet score, mean (SD)	6.9 (2.6)	24.5 (2.8)	<0.0001	6.4 (2.8)	24.1 (2.5)	<0.0001
Carbohydrate‐related nutrients and foods						
Carbohydrate (energy %), mean (SD)	48.8 (3.1)	36.6 (3.1)	0.0002	49.1 (3.8)	37.2 (2.8)	0.0004
Protein (energy %), mean (SD)	18.3 (2.4)	22.1 (3.1)	0.0005	17.6 (2.2)	21.2 (2.5)	0.001
Total fat (energy %), mean (SD)	33.3 (4.3)	41.5 (3.0)	0.0001	33.6 (4.3)	41.9 (2.5)	0.0004
Fibre (g/MJ), mean (SD)	22.4 (8.3)	19.1 (7.5)	0.003	22.7 (7.8)	20.0 (7.6)	0.008
Glycaemic index, mean (SD)	52.3 (3.9)	50.8 (4.3)	0.0007	53.5 (3.5)	52.6 (3.5)	0.21
Glycaemic load, mean (SD)	112.9 (41.5)	87.4 (35.2)	0.0009	122.9 (40.0)	101.8 (45.9)	0.11
Whole grains (serves), median (IQR)	2.5 (1.3–3.7)	1.9 (1.0–2.9)	0.04	2.3 (1.2–3.4)	1.5 (0.5–2.5)	0.004
Refined grains (serves), median (IQR)	1.7 (0.9–2.6)	1.2 (0.7–2.2)	0.05	2.0 (1.2–3.3)	1.9 (1.0–2.9)	0.26
Fruit juice (serves), median (IQR)	0.4 (0.1–1.2)	0.2 (0.1–0.6)	0.01	1.1 (0.3–1.9)	0.4 (0.1–1.1)	0.02
Fruit (serves), median (IQR)	1.7 (0.9–2.2)	1.0 (0.6–1.6)	0.02	1.2 (0.5–2.0)	1.0 (0.6–1.7)	0.25
Vegetables (serves), mean (SD)	2.0 (0.9)	2.1 (1.0)	0.07	2.1 (1.0)	2.2 (1.1)	0.05
Discretionary foods (serves), median (IQR)	2.8 (1.6–4.0)	2.7 (1.9–3.9)	0.09	2.9 (1.8–4.6)	3.0 (2.1–4.4)	0.03
Other nutrients and foods						
Total energy intake (kJ), mean (SD)	7396 (2596)	7823 (3007)	0.04	7837 (2389)	8673 (3750)	0.11
Saturated fat (energy %), mean (SD)	14.0 (2.7)	17.6 (2.6)	0.0009	14.2 (2.7)	18.4 (2.5)	0.003
Monounsaturated fat (energy %), mean (SD)	0.3 (0.1)	0.4 (0.1)	0.0004	0.3 (0.1)	0.4 (0.1)	0.0005
Polyunsaturated fat (energy %), mean (SD)	4.8 (1.6)	5.5 (1.7)	0.02	4.9 (1.6)	5.2 (1.8)	0.05
Dairy (serves), mean (SD)	1.9 (0.8)	1.9 (0.9)	0.003	2.0 (0.8)	2.0 (0.9)	0.65
Fish (serves), median (IQR)	0.1 (0.1–0.2)	0.1 (0.1–0.3)	0.001	0.1 (0.1–0.2)	0.1 (0.1–0.2)	0.27
Red meat (serves), median (IQR)	0.6 (0.4–1.0)	1.4 (0.9–2.1)	0.01	0.6 (0.3–1.0)	1.5 (1.1–2.3)	0.01
Processed meat (serves), median (IQR)	0.2 (0.1–0.4)	0.4 (0.2–0.7)	0.01	0.2 (0.1–0.4)	0.5 (0.3–0.8)	0.01

Abbreviations: IQR, interquartile range; SD, standard deviation.

^a^ Food group serving sizes are as per the 2013 Australian Dietary Guidelines (see Supporting Information: Table 1).

^b^ *p* Values from linear regression (*p* values comparing values across all quartiles; results for quartiles 2 and 3 are not reported to reduce the size of the table).

Among women who consumed a diet relatively low in carbohydrates (LCD score quartile 4), women attempting to lose weight had a lower GL, lower intake of total energy (−850 kJ) and refined grains (−0.7 serves) and a higher intake of whole grains (+0.4 serves), compared with women not attempting weight loss (Table 5).

#### 3.2.2 Women with recent GDM

For women with and without recent GDM, the distribution of dietary intake across quartiles of LCD was mostly similar, except for the consumption of vegetables and discretionary foods (Table 4). Consumption of these food groups did not differ by LCD score quartile among women without recent GDM, while women with GDM in their recent pregnancy who restricted their carbohydrate intake the most compared with the least had higher daily intake of vegetables (+0.9 serves) and lower daily intake of discretionary foods (−1.1 serves).

**Table 4:** Dietary intake of post‐partum women with and without a recent gestational diabetes diagnosis according to low carbohydrate‐diet score, *N*[weighted] = 1911.

Dietary intake^a^	Women with recent GDM, *N*[weighted] = 88			Women with no recent GDM, *N*[weighted] = 1823		
	Quartile 1 (least carbohydrate restriction), *N*[weighted] = 20 (23.1%)	Quartile 4 (most carbohydrate restriction), *N*[weighted] = 27 (30.2%)	*p* Value^b^	Quartile 1 (least carbohydrate restriction), *N*[weighted] = 591 (32.4%)	Quartile 4 (most carbohydrate restriction), *N*[weighted] = 397 (21.8%)	*p* Value^b^
Low carbohydrate‐diet score, mean (SD)	7.0 (1.8)	25.0 (2.7)	0.04	6.8 (2.6)	24.3 (2.7)	<0.0001
Carbohydrate‐related nutrients and foods						
Carbohydrate (energy %), mean (SD)	48.6 (2.7)	36.3 (3.3)	0.02	48.8 (3.5)	36.9 (2.9)	<0.0001
Protein (energy %), mean (SD)	18.6 (1.9)	22.2 (3.6)	0.02	18.1 (2.4)	21.9 (3.0)	0.0001
Total fat (energy %), mean (SD)	33.2 (4.3)	41.8 (2.3)	0.02	33.4 (4.3)	41.5 (2.9)	0.0002
Fibre (g/MJ), mean (SD)	22.4 (7.7)	18.5 (6.0)	0.17	22.5 (8.2)	19.3 (7.4)	0.01
Glycaemic index, mean (SD)	52.2 (2.4)	50.0 (4.5)	0.005	52.5 (3.8)	51.2 (4.2)	0.006
Glycaemic load, mean (SD)	115.8 (42.6)	81.5 (33.5)	0.03	115.2 (40.5)	90.3 (35.4)	0.009
Whole grains (serves), median (IQR)	2.6 (1.4–4.6)	1.7 (1.4–2.9)	0.05	2.4 (1.3–3.6)	1.8 (0.8–2.9)	0.03
Refined grains (serves), median (IQR)	1.4 (1.1–2.7)	0.9 (0.4–1.6)	0.55	1.7 (0.9–2.7)	1.4 (0.8–2.3)	0.08
Fruit juice (serves), median (IQR)	0.3 (0.1–0.9)	0.1 (0.0–0.5)	0.43	0.7 (0.1–1.4)	0.2 (0.1–0.8)	0.06
Fruit (serves), median (IQR)	1.5 (0.9–2.0)	0.9 (0.5–1.5)	0.23	1.6 (0.8–2.2)	1.0 (0.6–1.6)	0.05
Vegetables (serves), mean (SD)	1.4 (0.7)	2.3 (1.2)	0.04	2.0 (0.9)	2.1 (1.0)	0.13
Discretionary foods (serves), median (IQR)	3.0 (1.4–5.1)	1.9 (1.5–4.4)	0.02	2.8 (1.6–4.2)	2.9 (2.0–3.9)	0.85
Other nutrients and foods						
Total energy intake (kJ), mean (SD)	7630 (2750)	7376 (2573)	0.20	7503 (2505)	7969 (2985)	0.18
Saturated fat (energy %), mean (SD)	13.0 (2.9)	18.2 (2.4)	0.007	14.1 (2.7)	17.7 (2.6)	0.0001
Monounsaturated fat (energy %), mean (SD)	0.3 (0.1)	0.4 (0.1)	0.04	0.3 (0.1)	0.4 (0.1)	0.0002
Polyunsaturated fat (energy %), mean (SD)	5.8 (1.8)	5.1 (1.6)	0.21	4.8 (1.5)	5.5 (1.8)	0.004
Dairy (serves), mean (SD)	2.1 (0.8)	1.9 (0.9)	0.21	1.9 (0.8)	1.9 (0.9)	0.19
Fish (serves), median (IQR)	0.1 (0.1–0.2)	0.2 (0.1–0.3)	0.61	0.1 (0.1–0.2)	0.1 (0.1–0.2)	0.0006
Red meat (serves), median (IQR)	0.5 (0.3–1.0)	1.1 (0.9–1.6)	0.04	0.6 (0.3–1.0)	1.4 (1.0–2.1)	0.01
Processed meat (serves), median (IQR)	0.2 (0.1–0.4)	0.3 (0.2–0.6)	0.03	0.2 (0.1–0.4)	0.4 (0.3–0.7)	0.003

Abbreviations: GDM, gestational diabetes; IQR, interquartile range; SD, standard deviation.

^a^ Food group serving sizes are as per the 2013 Australian Dietary Guidelines (see Supporting Information: Table 1).

^b^ *p* Values from linear regression (*p* values comparing values across all quartiles; results for quartiles 2 and 3 are not reported to reduce the size of the table).

Among women who consumed a diet relatively low in carbohydrates (LCD score quartile 4), women with recent GDM had a lower intake of refined grains (−0.5 serves), while intake of all other nutrients and food groups did not differ (Table 5).

**Table 5:** Dietary intake of post‐partum women who consume a diet relatively low in carbohydrates (low carbohydrate‐diet score in quartile 4) according to weight loss attempt (*N*[weighted] = 2056) and recent gestational diabetes diagnosis (*N*[weighted] = 1911).

Dietary intake^a^	Women attempting weight loss, *N*[weighted] = 338	Women not attempting weight loss, *N*[weighted] = 130	*p* Value^a^	Women with recent GDM, *N*[weighted] = 27	Women with no recent GDM, *N*[weighted] = 397	*p* Value^b^
Low carbohydrate‐diet score, mean (SD)	24.5 (2.6)	24.1 (2.6)	0.14	25.1 (2.5)	24.3 (2.6)	0.06
Carbohydrate‐related nutrients and foods						
Carbohydrate (energy %), mean (SD)	36.6 (3.0)	37.2 (2.7)	0.06	36.3 (3.1)	36.9 (2.8)	0.0005
Protein (energy %), mean (SD)	22.2 (3.0)	21.2 (2.5)	0.004	22.2 (3.3)	21.9 (2.9)	0.50
Total fat (energy %), mean (SD)	41.5 (2.9)	41.9 (2.4)	0.35	41.8 (2.2)	41.5 (2.8)	0.56
Fibre (g/MJ), mean (SD)	19.1 (7.1)	20.0 (7.5)	0.44	18.5 (5.6)	19.3 (7.2)	0.06
Glycaemic index, mean (SD)	50.8 (4.1)	52.6 (3.5)	0.006	50.0 (4.2)	51.2 (4.1)	0.21
Glycaemic load, mean (SD)	87.4 (33.6)	101.8 (45.5)	0.02	81.5 (31.3)	90.3 (34.2)	0.17
Whole grains (serves), median (IQR)	1.9 (1.0–2.9)	1.5 (0.5–2.5)	0.002	1.7 (1.4–2.9)	1.8 (0.8–2.9)	0.15
Refined grains (serves), median (IQR)	1.2 (0.7–2.2)	1.9 (1.0–2.8)	0.005	0.9 (0.4–1.6)	1.4 (0.8–2.3)	0.02
Fruit juice (serves), median (IQR)	0.2 (0.1–0.6)	0.4 (0.1–1.1)	0.0006	0.1 (0.1–0.5)	0.2 (0.1–0.8)	0.30
Fruit (serves), median (IQR)	1.0 (0.6–1.6)	1.0 (0.6–1.7)	0.76	0.9 (0.5–1.5)	1.0 (0.6–1.6)	0.002
Vegetables (serves), mean (SD)	2.1 (1.0)	2.2 (1.1)	0.32	2.3 (1.1)	2.1 (1.0)	0.009
Discretionary foods (serves), median (IQR)	2.7 (1.9–3.9)	3.0 (2.1–4.4)	0.11	1.9 (1.5–4.4)	2.9 (2.0–3.9)	0.28
Other nutrients and foods						
Total energy intake (kJ), mean (SD)	7823 (2869)	8673 (3712)	0.07	7376 (2402)	7969 (2888)	0.19
Saturated fat (energy %), mean (SD)	17.6 (2.5)	18.4 (2.4)	0.02	18.2 (2.3)	17.7 (2.5)	<0.0001
Monounsaturated fat (energy %), mean (SD)	0.4 (0.1)	0.4 (0.1)	0.29	0.4 (0.1)	0.4 (0.1)	0.93
Polyunsaturated fat (energy %), mean (SD)	5.5 (1.7)	5.2 (1.8)	0.03	5.1 (1.4)	5.5 (1.7)	0.16
Dairy (serves), mean (SD)	1.9 (0.8)	2.0 (0.9)	0.57	1.9 (0.8)	1.9 (0.8)	0.31
Fish (serves), median (IQR)	0.1 (0.1–0.2)	0.1 (0.1–0.2)	0.65	0.2 (0.1–0.3)	0.1 (0.1–0.2)	0.81
Red meat (serves), median (IQR)	1.4 (0.9–2.1)	1.5 (1.1–2.3)	0.15	1.1 (0.9–1.6)	1.4 (1.0–2.1)	0.14
Processed meat (serves), median (IQR)	0.4 (0.2–0.7)	0.5 (0.3–0.8)	0.01	0.3 (0.2–0.6)	0.4 (0.3–0.7)	0.12

Abbreviations: GDM, gestational diabetes; SD, standard deviation.

^a^ Food group serving sizes are as per the 2013 Australian Dietary Guidelines (see Supporting Information: Table 1).

^b^ *p* Values from linear regression.

## 4 DISCUSSION

In this national population‐based study, we observed that the dietary intake of post‐partum women who consumed a diet relatively low in carbohydrates (37%) was characterised by both better and worse food choices when compared with women with a diet relatively high in carbohydrates (49%). Overall, women with the lowest relative carbohydrate intake consumed less refined grains (−0.4 serves/day) and fruit juice (−0.5), but also less whole grains (−0.6) and fruit (−0.6) and more red meat (+0.8) and processed meat (+0.3), compared with women with the highest relative carbohydrate intake. Different food groups were restricted depending on whether women were attempting weight loss or had been diagnosed with GDM in their recent pregnancy.

A limited number of previous studies have examined the dietary intake and quality of women of childbearing age who were restricting their carbohydrate intake (Bao et al., 2016; Looman et al., 2018; Tang et al., 2021). In line with our findings, among 4502 women with a history of GDM from the US Nurses’ Health Study II, a diet relatively low in carbohydrates (top vs. bottom quintile of LCD score: 42% vs. 57% carbohydrates, 22% vs. 17% protein and 37% vs. 27% total fat) was characterised by higher consumption of red meat, and lower consumption of fruit, vegetables and whole grains (Bao et al., 2016). Similarly, among 500 post‐partum women recruited from a hospital in Guangzhou, China during 2017 and 2018, women who restricted their carbohydrate intake the most (top vs. bottom tertile of LCD score: 31% vs. 50% carbohydrates, 22% vs. 17% protein and 47% vs. 34% total fat) reported lower consumption of grains and fruits, and higher intake of red and processed meat (Tang et al., 2021). Collectively, these findings from studies of Chinese, United States and Australian populations suggest that women who restrict their carbohydrate intake do so by reducing consumption of core food groups including grains and fruit, and have a diet higher in animal‐source foods. While in our study we also observed positive aspects of an LCD including lower consumption of refined grains and fruit juice, women who wish to restrict their carbohydrate intake may lack knowledge about dietary guidelines and carbohydrates (Churuangsuk et al., 2020). Increasing their knowledge and supporting and enabling women to consume a balanced diet may therefore improve diet quality in line with recommendations for post‐partum women.

Consistent with the popular belief that LCDs are an effective strategy for weight loss (Churuangsuk et al., 2020; Crowe & Cameron‐Smith, 2005; International Food Information Council, 2019), the proportion of women in our study who were attempting to lose weight was higher in the top versus bottom quartile of the LCD score (most vs. least carbohydrate restriction). There is however limited evidence on the benefits of an LCD for post‐partum weight loss (Alderete et al., 2020; Chen et al., 2021; Castro et al., 2019; Vincze et al., 2019). While the best approach to weight loss for post‐partum women is unknown and further research is needed (Dodd et al., 2018; Vincze et al., 2019), women should be educated on the potential negative effects of restricting core food groups considered to be high in carbohydrates (such as whole grains and fruit) while increasing intake of high‐protein and fat animal foods (such as red meat and processed meat). Even though women who consumed a LCD and who were attempting weight loss had a higher intake of whole grains compared with women consuming a LCD and not attempting weight loss, their whole grain consumption was still lower compared with women consuming a diet with a relative carbohydrate content in line with dietary guidelines (National Health and Medical Research Council, 2013). Adequately supporting and enabling women to achieve their weight goals may reduce the negative impact of PPWR, overweight and obesity on future pregnancies (Dodd et al., 2018; Schoenaker et al., 2020) as well as the lifelong maternal risk of chronic diseases such as diabetes and cardiovascular disease (Jacob et al., 2017; Poston et al., 2016).

Post‐partum women with recent GDM were also more likely to restrict their carbohydrate intake in our study. This is in line with findings from limited previous studies that women with GDM reduce their carbohydrate intake following GDM diagnosis (Hinkle et al., 2021), may continue to restrict their carbohydrate intake following pregnancy (Tang et al., 2021) and have poor diet quality, especially in terms of fruit and grain intake (Morrison et al., 2012). The effects of carbohydrate restriction after GDM on future type 2 diabetes risk are unclear. A recent systematic review found that while dietary intervention studies generally indicated a trend towards the beneficial effects of reducing carbohydrate intake, these studies had a high risk of bias (D'Arcy et al., 2020). Observational studies have shown poorer diabetes outcomes for women with a high animal fat and protein LCD, and better outcomes for women consuming a high plant fat and protein LCD including diets rich in fruit, vegetables, nuts, fish and legumes, and low in red and processed meats and sugar‐sweetened beverages (D'Arcy et al., 2020). Women with recent GDM should therefore be advised to eat healthily in line with general dietary guidelines (D'Arcy et al., 2020; National Health and Medical Research Council, 2013). Encouragingly, women in our study with recent GDM who restricted their carbohydrates the most had higher vegetable intake and lower discretionary food intake than those who consumed more carbohydrates, while these foods were not related to carbohydrate restriction among those without GDM in their recent pregnancy. Also, women who consumed a LCD and who had GDM in the recent pregnancy had a lower intake of refined grains and fruit juice compared with women consuming a LCD who did not have GDM in their recent pregnancy. This may reflect the education women with previous GDM received during pregnancy from health professionals, and their higher level of knowledge of dietary guidelines and carbohydrate quality.

The most recent National Health and Medical Research Council (2013) recommend 45%–65% of total energy from carbohydrates, 15%–25% from protein and 20%–35% from total fat (National Health and Medical Research Council, 2013). These recommendations are in line with the macronutrient distribution observed in the bottom LCD score quartile in our study (49% of total energy from carbohydrates, 18% from proteins and 33% from total fats). However, the daily intake of core food groups (e.g., fruit, vegetables, grains, dairy, and meat and alternatives) among women in this quartile remains poorly aligned with recommended intakes in the Australian Dietary Guidelines, as also previously described for both pregnant and nonpregnant women in the ALSWH (Mishra et al., 2015). Optimal dietary intake and quality are important for post‐partum women to reduce PPWR, maternal obesity and risks in subsequent pregnancies, as well as diet‐related long‐term chronic disease development (Chen et al., 2021; D'Arcy et al., 2020; Luke et al., 2016; Schoenaker et al., 2020; Tang et al., 2021). Moreover, diet quality is important for post‐partum women who are breastfeeding, and women on LCDs should be aware of the rare but dangerous potential of lactation ketoacidosis (Osborne & Oliver, 2022). Health professionals could have an important role in providing advice and support to improve the overall diet quality of post‐partum women (Churuangsuk et al., 2020; Morrison et al., 2012; Teh et al., 2021); for example, at routine health checks such as (in the Australian context) the 6–8‐week post‐natal health check with a midwife, family doctor or obstetrician, or as part of the Maternal and Child Health service at 10 key stages through to child age 3.5 years.

Findings from this study add to the limited evidence on the dietary intake and quality of post‐partum women who restrict their carbohydrate intake and are based on a large and broadly representative sample of Australian women (Brown et al., 1998; Dobson et al., 2015; Lee et al., 2005). Some limitations should be considered when interpreting the results, including measurement errors in dietary intake despite the use of a validated FFQ. Moreover, the LCD score is based on the distribution of macronutrient intake in our study population with women in the highest quartile (most carbohydrate restriction) still consuming 37% of total energy from carbohydrates. The dietary intake of women with more extreme carbohydrate restriction (such as a typical ketogenic diet with 5%–10% of energy from carbohydrates) could therefore not be described. Diet quality was described in terms of core food groups and key nutrients, and no data were available on the use of specific dietary supplements. Also, no data were available on whether women intentionally restricted their carbohydrate intake and their reasons for doing so. Differences in dietary intake by LCD score were generally small (although statistically significant), however, differences in daily intake of refined and whole grains, fruit and fruit juice, and red and processed meat are clinically relevant at a population level over time. Lastly, the dietary data in our study were collected in 2003 and 2009, and further research in more contemporary populations of post‐partum women is needed to inform up‐to‐date public health messages.

In conclusion, findings from this cross‐sectional population‐based cohort study suggest that post‐partum women who consume a diet relatively low in carbohydrates may restrict both healthy and unhealthy core food groups. Given the popularity of LCDs for weight loss and the critical role of carbohydrate quantity and quality in chronic disease prevention, health professionals should inform and support women who wish to restrict their carbohydrate intake to consume a balanced diet in line with dietary guidelines, including adequate intake of whole grains and fruit.

## AUTHOR CONTRIBUTIONS

Danielle A. J. M. Schoenaker conceptualised the idea and designed the research. Sophie Lewandowski and Danielle A. J. M. Schoenaker performed the statistical analysis, drafted the manuscript and had primary responsibility for the final content of the manuscript. All authors contributed to the interpretation of the results, provided important intellectual content, edited the manuscript and approved the final version for submission.

## CONFLICT OF INTEREST STATEMENT

The authors declare no conflict of interest.

## Supporting information

Supporting information.

## Data Availability

Data are available from the Australian Longitudinal Study on Women's Health (contact ALSWH Data and Analytic Services at www.alswh.org.au/) for researchers who meet the criteria for access to confidential data.
