# Supplementary material for: Quality of low‐carbohydrate diets among Australian post‐partum women: Cross‐sectional analysis of a national population‐based cohort study
Source: Matern Child Nutr. 2023 Mar 20;19(3):e13502. doi: 10.1111/mcn.13502 (PMC10262910; doi:10.1111/mcn.13502)
Supplement: Supplementary file 1 — Supporting information. [file MCN-19-e13502-s001.docx]

**SUPPLEMENTARY MATERIAL**

**Supplementary Table 1.** Description of food items and serving sizes used to create food groups

| Food group | Serving size**^†^** | Food Frequency Questionnaire food items included |
| --- | --- | --- |
| Whole grains | 40g | Wholemeal bread, high-fibre white bread, multi-grain bread and rye bread |
|  | 30g | All bran, bran flakes, Weet-Bix and muesli |
|  | 120g | Porridge |
| Refined grains | 40g | White bread |
|  | 100g | Pasta and rice |
|  | 30g | Cornflakes |
|  | 35g | Crackers |
| Fruit juice | 125g**^‡^** | Fruit juice |
| Fruit | 150g | Oranges, apples, pears, bananas, melon, pineapple, strawberries, apricots, peaches, mangoes, tinned fruit |
|  | 45g | Avocado |
| Vegetables | 75g | Bean sprouts, beetroot, broccoli, cabbage, carrot, cauliflower, capsicum, cucumber, celery, garlic, green beans, mushrooms, onion, peas, pumpkin, spinach, tomatoes, zucchini, potatoes |
| Dairy | 250g | Full cream milk, reduced fat milk, skim milk and soy milk |
|  | 200g | Yoghurt |
|  | 120g | Ricotta or cottage cheese |
|  | 40g | Hard cheese, firm cheese, soft cheese and low-fat cheese |
| Fish | 100g | Fish (steamed/grilled/baked/tinned) |
| Red and processed meat | 65g | Beef, veal, lamb, pork |
|  | 80g | Bacon |
|  | 60g | Ham, salami, sausages |
| Discretionary foods | 75g | Ice cream |
|  | 25g | Sweet biscuits and chocolate |
|  | 4g | Added sugar |
|  | 60g | Jam, meat pies, pizza, hamburgers |
|  | 40g | Cakes |
|  | 200g | Flavoured milk drink |
|  | 30g | Crisps |
|  | 20g | Butter, margarine, butter/margarine blend, polyunsaturated margarine, monounsaturated margarine |

**^†^** Serving sizes according to the Australian Dietary Guidelines 2013

**^‡^** For milk, flavoured milk and fruit juice, 1g was assumed to equal 1ml
